# Supplementary material for: A novel lineage of candidate pheromone receptors for sex communication in moths
Source: eLife. 2019 Dec 10;8:e49826. doi: 10.7554/eLife.49826 (PMC6904214; doi:10.7554/eLife.49826)
Supplement: Supplementary file 1. [file elife-49826-supp1.docx]

**Supplementary File 1.** List of synthetic compounds used for electrophysiology experiments.

| Full Name | Abbreviation | CAS | Source | Purity |
| --- | --- | --- | --- | --- |
| (*Z*)-5-decenyl acetate | (*Z*)5-10:OAc | 67446-07-5 | Sigma | 95% |
| dodecyl acetate | 12:OAc | 112-66-3 | *Synthesized in the lab* | 99% |
| (*E*)-7-dodecenyl acetate | (*E*)7-12:OAc | 16677-06-8 | *Synthesized in the lab* | 95% |
| (*Z*)-7-dodecenyl acetate | (*Z*)7-12:OAc | 14959-86-5 | Sigma | 95% |
| (*Z*)-7-dodecen-1-ol | (*Z*)7-12:OH | 20056-92-2 | *Synthesized in the lab* | 95% |
| (*Z*)-9-dodecenyl acetate | (*Z*)9-12:OAc | 16974-11-1 | *Synthesized in the lab* | 95% |
| (*Z,E*)-7,9-dodecadienyl acetate | (*Z,E*)-7,9-12:OAc | 55774-32-8 | *Synthesized in the lab* | 95% |
| 11-dodecenyl acetate | ∆11-12:OAc | 35153-10-7 | *Synthesized in the lab* | 95% |
| tetradecyl acetate | 14:OAc | 638-59-5 | *Synthesized in the lab* | 99% |
| (*Z*)-9-tetradecenyl acetate | (*Z*)9-14:OAc | 16725-53-4 | *Synthesized in the lab* | 95% |
| (*Z*)-9-tetradecen-1-ol | (*Z*)9-14:OH | 35153-15-2 | *Synthesized in the lab* | 98% |
| (*Z*)-9-tetradecenal | (*Z*)9-14:Al | 53939-27-8 | *Synthesized in the lab* | 96% |
| (*E*)-11-tetradecenyl acetate | (*E*)11-14:OAc | 33189-72-9 | *Synthesized in the lab* | 95% |
| (*Z,E*)-9,11-tetradecadienyl acetate | (*Z,E*)-9,11-14:OAc | 50767-79-8 | *Synthesized in the lab* | 94% |
| (*Z,E*)-9,11-tetradecadien-1-ol | (*Z,E*)-9,11-14:OH | 63025-02-5 | Pherobank | 96% |
| (*Z*)-11-tetradecenyl acetate | (*Z*)11-14:OAc | 20711-10-8 | *Synthesized in the lab* | 96% |
| (*Z,Z*)-9,11-tetradecadienyl acetate | (*Z,Z*)-9,11-14:OAc | 54664-98-1 | *Synthesized in the lab* | 95% |
| (*E,E*)-9,12-tetradecadienyl acetate | (*E,E*)-9,12-14:OAc | - | Pherobank | 98% |
| (*Z,E*)-9,12-tetradecadienyl acetate | (*Z,E*)-9,12-14:OAc | 30507-70-1 | *Synthesized in the lab* | 96% |
| (*Z,E*)-9,12-tetradecadien-1-ol | (*Z,E*)-9,12-14:OH | 51937-00-9 | *Synthesized in the lab* | 92% |
| (*E,E*)-10,12-tetradecadienyl acetate | (*E,E*)-10,12-14:OAc | 69775-61-7 | *Synthesized in the lab* | 95% |
| (*Z,Z*)-9,12-tetradecadienyl acetate | (*Z,Z*)-9,12-14:OAc | 51354-22-4 | Pherobank | 98% |
| (*Z*)-9-hexadecenal | (*Z*)9-16:Al | 56219-04-6 | Pherobank | 96% |
| (*Z*)-11-hexadecenyl acetate | (*Z*)11-16:OAc | 34010-21-4 | *Synthesized in the lab* | 95% |
| (*Z*)-11-hexadecen-1-ol | (*Z*)11-16:OH | 56683-54-6 | *Synthesized in the lab* | 95% |
| (*Z*)-11-hexadecenal | (*Z*)11-16:Al | 53939-28-9 | Pherobank | 97% |
| benzyl alcohol |  | 100-51-6 | Sigma | 99% |
| (±)-linalool |  | 78-70-6 | Sigma | 97% |
