# Supplementary material for: A novel lineage of candidate pheromone receptors for sex communication in moths
Source: eLife. 2019 Dec 10;8:e49826. doi: 10.7554/eLife.49826 (PMC6904214; doi:10.7554/eLife.49826)
Supplement: Supplementary file 2. [file elife-49826-supp2.docx]

**Supplementary File 2.** Functional and sex-biased expression data available for lepidopteran pheromone receptors (as of September 2018).

| Species | Gene | Best ligand(s) | Expression method | Ref. | Expression bias | Quantification method | Ref. |
| --- | --- | --- | --- | --- | --- | --- | --- |
| *Agrotis ipsilon* | AipsOR1 |  |  |  | Male | RNAseq, qPCR | (1) |
|  | AipsOR3 |  |  |  | Male | RNAseq, qPCR | (1) |
|  | AipsOR4 |  |  |  | Male | RNAseq, qPCR | (1) |
| *Agrotis segetum* | AsegOR1 | Z5-10:OAc; Z7-12:OAc; Z8-12:OAc | *Xenopus* oocytes | (2) |  |  |  |
|  | AsegOR3 | Z3,Z6,Z9-21:H | *Xenopus* oocytes | (3) |  |  |  |
|  | AsegOR4 | Z7-12:OAc | *Xenopus* oocytes | (2) |  |  |  |
|  | AsegOR5 | Z9-14:OAc | *Xenopus* oocytes | (2) |  |  |  |
|  | AsegOR6 | Z5-10:OH | *Xenopus* oocytes | (2) |  |  |  |
|  | AsegOR7 | Z5-10:OAc | *Xenopus* oocytes | (2) |  |  |  |
|  | AsegOR8 | Z5-10:OAc; Z5-10:OH | *Xenopus* oocytes | (2) |  |  |  |
|  | AsegOR9 | Z5-10:OAc | *Xenopus* oocytes | (2) |  |  |  |
|  | AsegOR10 | Z9-14:OAc | *Xenopus* oocytes | (2) |  |  |  |
| *Antherea polyphemus* | ApolOR1 | E6,Z11:Ald | HEK293 cells | (4) |  |  |  |
| *Amyelois transitella* | AtraOR1 | Z11,Z13-16:Ald | *Xenopus* oocytes | (5) |  |  |  |
|  | AtraOR3 | Z11-16:Ald | *Xenopus* oocytes | (5) |  |  |  |
| *Athethis dissimilis* | AdisOR3 |  |  |  | Male | RNAseq | (6) |
|  | AdisOR6 |  |  |  | Male | RNAseq | (6) |
|  | AdisOR14 |  |  |  | Male | RNAseq | (6) |
|  | AdisOR17 |  |  |  | Female | RNAseq | (6) |
|  | AdisOR32b |  |  |  | Female | RNAseq | (6) |
| *Bombyx mori* | BmorOR1 | E10,Z12-16:OH | *Xenopus* oocytes | (7) | Male | qPCR | (8) |
|  | BmorOR3 | E10,Z12-16:Ald | *Xenopus* oocytes | (9) | Male | qPCR | (8) |
|  | BmorOR4 |  |  |  | Male | qPCR | (8) |
|  | BmorOR5 |  |  |  | Male | qPCR | (8) |
|  | BmorOR6 |  |  |  | Male | qPCR | (8) |
|  | BmorOR24 | 2-hexenyl acetate | *Xenopus* oocytes | (10) |  |  |  |
|  | BmorOR30 |  |  |  | Female | qPCR | (8) |
|  | BmorOR42 | (±)-linalool | *Xenopus* oocytes | (10) |  |  |  |
| *Conogethes punctiferalis* | CpunOR22 |  |  |  | Male | RNAseq, qPCR | (11) |
|  | CpunOR26 |  |  |  | Male | RNAseq, qPCR | (11) |
|  | CpunOR46 |  |  |  | Male | RNAseq, qPCR | (11) |
| *Ctenopseustis herana* | CherOR7 | Z8-14:OAc | HEK293 cells | (12) | Male | RNAseq, qPCR | (12) |
|  | CherOR30 |  |  |  | Male | RNAseq, qPCR | (12) |
| *Ctenopseustis obliquana* | CoblOR7 | Z8-14:OAc | HEK293 cells | (12) | Male | RNAseq, qPCR | (12) |
|  | CoblOR30 |  |  |  | Male | RNAseq, qPCR | (12) |
| *Cydia pomonella* | CpomOR1 |  |  |  | Male | RNAseq | (13) |
|  | CpomOR3 | pear ester | HEK293 cells, *Drosophila* OSNs | (14) |  |  |  |
|  | CpomOR5 |  |  |  | Male | RNAseq | (13) |
|  | CpomOR6 | E8,E10-12:OH | HEK293 cells, *Drosophila* OSNs | (14) | Male | RNAseq | (13) |
|  | CpomOR7 |  |  |  | Male | RNAseq | (13) |
|  | CpomOR21 |  |  |  | Female | RNAseq | (13) |
|  | CpomOR22 |  |  |  | Female | RNAseq | (13) |
|  | CpomOR30 |  |  |  | Female | RNAseq | (13) |
|  | CpomOR31 |  |  |  | Male | RNAseq | (13) |
|  | CpomOR41 |  |  |  | Female | RNAseq | (13) |
| *Dendrolimus punctatus* | DpunOR4 |  |  |  | Female | RNAseq, qPCR | (15) |
|  | DpunOR7 |  |  |  | Male | RNAseq, qPCR | (15) |
|  | DpunOR20 |  |  |  | Male | RNAseq, qPCR | (15) |
|  | DpunOR45 |  |  |  | Male | RNAseq, qPCR | (15) |
|  | DpunOR46 |  |  |  | Male | RNAseq, qPCR | (15) |
|  | DpunOR47 |  |  |  | Female | RNAseq, qPCR | (15) |
|  | DpunOR49 |  |  |  | Female | RNAseq, qPCR | (15) |
|  | DpunOR51 |  |  |  | Male | RNAseq, qPCR | (15) |
|  | DpunOR54 |  |  |  | Male | RNAseq, qPCR | (15) |
|  | DpunOR63 |  |  |  | Male | RNAseq, qPCR | (15) |
|  | DpunOR67 |  |  |  | Female | RNAseq, qPCR | (15) |
|  | DpunOR68 |  |  |  | Female | RNAseq, qPCR | (15) |
| *Diaphania indica* | DindOR1 | E11-16:Ald | *Xenopus* oocytes | (16) |  |  |  |
| *Ectropis grisescens* | EgriOR24 |  |  |  | Male | RNAseq | (17) |
|  | EgriOR28 |  |  |  | Male | RNAseq | (17) |
|  | EgriOR31 | Z3,Z6,Z9-18:H | *Xenopus* oocytes | (17) | Male | RNAseq | (17) |
|  | EgriOR37 |  |  |  | Male | RNAseq | (17) |
|  | EgriOR44 |  |  |  | Male | RNAseq | (17) |
| *Epiphyas postvittana* | EposOR1 | methyl salicylate | Sf9 cells | (18) |  |  |  |
|  | EposOR3 | citral | Sf9 cells | (18) |  |  |  |
|  | EposOR6 |  |  |  | Male | RNAseq, qPCR | (19) |
|  | EposOR7 |  |  |  | Male | RNAseq, qPCR | (19) |
|  | EposOR30 |  |  |  | Male | RNAseq, qPCR | (19) |
|  | EposOR31 |  |  |  | Female | RNAseq, qPCR | (19) |
|  | EposOR33 |  |  |  | Female | RNAseq, qPCR | (19) |
|  | EposOR34 |  |  |  | Male | RNAseq, qPCR | (19) |
|  | EposOR36 |  |  |  | Female | RNAseq, qPCR | (19) |
|  | EposOR40 |  |  |  | Female | RNAseq, qPCR | (19) |
| *Eriocrania semipurpurella* | EsemOR1 | β-caryophyllene | HEK293 cells | (20) |  |  |  |
|  | EsemOR3 | (2S,6Z)-6-nonen-2-ol | HEK293 cells | (20) |  |  |  |
|  | EsemOR5 | Z6-nonen-2-one | HEK293 cells | (20) |  |  |  |
| *Heliconius melpomene* | HmelOR5 |  |  |  | Female | RNAseq | (21) |
|  | HmelOR46 |  |  |  | Female | RNAseq | (21) |
|  | HmelOR70 |  |  |  | Female | RNAseq | (21) |
| *Helicoverpa armigera* | HarmOR6 | Z9-14:Ald; Z9-16:Ald | *Xenopus* oocytes | (22) |  |  |  |
|  | HarmOR12 | 3,7-dimethyl-2,6-octadienal | *Xenopus* oocytes | (23) |  |  |  |
|  | HarmOR13 | Z11-16:Ald | *Xenopus* oocytes | (22) | Male | qPCR | (22) |
|  | HarmOR14a |  |  |  | Male | qPCR | (22) |
|  | HarmOR14b | Z9-14:Ald | *Xenopus* oocytes | (24) | Male | qPCR | (24) |
|  | HarmOR15 |  |  |  | Male | qPCR | (22) |
|  | HarmOR16 | Z11-16:OH; Z9-16:Ald | *Xenopus* oocytes | (22) | Male | qPCR | (22) |
|  | HarmOR52 | 1-pentanol | *Xenopus* oocytes | (23) |  |  |  |
| *Helicoverpa assulta* | HassOR6 | Z9-16:OH | *Xenopus* oocytes | (24) | Male | qPCR | (24) |
|  | HassOR13 | Z11-16:Ald | *Xenopus* oocytes | (24) | Male | qPCR | (24) |
|  | HassOR14b |  |  |  | Male | qPCR | (24) |
|  | HassOR16 | Z9-14:Ald | *Xenopus* oocytes | (24) |  |  |  |
| *Heliothis virescens* | HvirOR6 | Z9-14:Ald | *Xenopus* oocytes | (25) |  |  |  |
|  | HvirOR13 | Z11-16:Ald | *Xenopus* oocytes | (25) | Male | qPCR | (26) |
|  | HvirOR14 | Z11-16:OAc | *Xenopus* oocytes | (25) | Male | qPCR | (26) |
|  | HvirOR16 | Z11-16:OH | *Xenopus* oocytes | (25) |  |  |  |
| *Lampronia capitella* | LcapOR3 |  |  |  | Female | RNAseq | (27) |
|  | LcapOR6 | Z9,Z11-14:Ald | HEK293 cells | (27) | Male | RNAseq | (27) |
|  | LcapOR7 | Z11-14:OH; Z11-14:OAc; Z11-14:Ald | HEK293 cells | (27) | Male | RNAseq | (27) |
|  | LcapOR8 | Z9,Z11-14:OH | HEK293 cells | (27) | Male | RNAseq | (27) |
|  | LcapOR28 |  |  |  | Female | RNAseq | (27) |
| *Manduca sexta* | MsexOR1 | E10,Z12-16:Ald | HEK293 and CHO cells | (28) | Male | RNAseq | (29) |
|  | MsexOR4 |  |  |  | Male | RNAseq | (29) |
|  | MsexOR15 |  |  |  | Female | RNAseq | (29) |
|  | MsexOR51 |  |  |  | Male | RNAseq | (29) |
|  | MsexOR83 |  |  |  | Male | RNAseq | (29) |
|  | MsexOR86 |  |  |  | Female | RNAseq | (29) |
| *Mythimna separata* | MsepOR1 | Z11-16:OAc | *Xenopus* oocytes | (16) |  |  |  |
| *Ostrinia furnacalis* | OfurOR3 | E12-14:OAc; Z12-14:OAc | *Xenopus* oocytes | (30) | Male | RNAseq, qPCR | (31) |
|  | OfurOR4 | Z12-14:OAc | *Xenopus* oocytes | (32) | Male | RNAseq, qPCR | (31) |
|  | OfurOR5a |  |  |  | Male | RNAseq, qPCR | (31) |
|  | OfurOR5b | Z9-14:OAc; Z11-14:OAc; E11-14:OAc; Z12-14:OAc | *Xenopus* oocytes | (32) | Male | RNAseq, qPCR | (31) |
|  | OfurOR6 | E12-14:OAC | *Xenopus* oocytes | (32) | Male | RNAseq, qPCR | (31) |
|  | OfurOR7 | Z9-14:OAc | *Xenopus* oocytes | (32) |  |  |  |
|  | OfurOR8 | E11-14:OAc; Z11-14:OAc | *Xenopus* oocytes | (32) | Male | RNAseq, qPCR | (31) |
|  | OfurOR53 |  |  |  | Female | RNAseq, qPCR | (31) |
| *Ostrinia latipennis* | OlatOR1 | E11-14:OH | *Xenopus* oocytes | (33) |  |  |  |
| *Ostrinia nubilalis* | OnubOR1 | E12-14:OAC | *Xenopus* oocytes | (34) | Male | qPCR | (34) |
|  | OnubOR3 | E12-14:OAc | *Xenopus* oocytes | (34) | Male | qPCR | (34) |
|  | OnubOR5 | E11-14:OAc; E12-14:OAc | *Xenopus* oocytes | (34) | Male | qPCR | (34) |
|  | OnubOR6 | Z11-14:OAc | *Xenopus* oocytes | (34) | Male | qPCR | (34) |
| *Ostrinia scapulalis* | OscaOR1 | E11-14:OH | *Xenopus* oocytes | (33) |  |  |  |
|  | OscaOR3 | Z9-14:OAc; Z11-14:OAc; E11-14:OAc | *Xenopus* oocytes | (35) |  |  |  |
|  | OscaOR4 | E11-14:OAc | *Xenopus* oocytes | (35) |  |  |  |
| *Operophtera brumata* | ObruOR1 | 1,Z3,Z6,Z9-19:H | *Xenopus* oocytes | (3) | Male | qPCR | (3) |
| *Planotortrix excessana* | PexcOR7 |  |  |  | Male | RNAseq, qPCR | (36) |
|  | PexcOR30 |  |  |  | Male | RNAseq, qPCR | (36) |
| *Planotortrix octo* | PoctOR7 |  |  |  | Male | RNAseq, qPCR | (36) |
|  | PoctOR30 |  |  |  | Male | RNAseq, qPCR | (36) |
| *Plutella xylostella* | PxylOR6 |  |  |  | Male | qPCR | (37) |
|  | PxylOR48 | Z9,E12-14:OAc; Z9-14:OAc | *Xenopus* oocytes | (38) | Male | qPCR | (38) |
|  | PxylOR49 | Z9-14:Ac; Z9-14:OH; Z9-14:Ald | *Xenopus* oocytes | (37) | Male | qPCR | (37) |
|  | PxylOR50 |  |  |  | Female | qPCR | (37) |
|  | PxylOR59 | Z11-16:Ald | *Xenopus* oocytes | (38) | Male | qPCR | (38) |
| *Sesamia inferens* | SinfOR21 | Z11-16:OH | *Xenopus* oocytes | (39) |  |  |  |
|  | SinfOR27 | Z9,E12-14:OAc | *Xenopus* oocytes | (39) |  |  |  |
|  | SinfOR29 | Z11-16:OAc | *Xenopus* oocytes | (39) |  |  |  |
| *Spodoptera exigua* | SexiOR3 | (E)-β-farnesene | *Xenopus* oocytes | (40) |  |  |  |
|  | SexiOR6 |  |  |  | Male | qPCR | (41) |
|  | SexiOR11 |  |  |  | Male | qPCR | (41) |
|  | SexiOR13 | Z9,E12-14:OAc; Z9-14:OAc | *Xenopus* oocytes | (42) | Male | qPCR | (41) |
|  | SexiOR16 | Z9-14:OH | *Xenopus* oocytes | (42) | Male | qPCR | (41) |
|  | SexiOR18 |  |  |  | Female | qPCR | (41) |
|  | SexiOR28 |  |  |  | Female | qPCR | (41) |
|  | SexiOR34 |  |  |  | Female | qPCR | (41) |
|  | SexiOR40 |  |  |  | Female | qPCR | (41) |
| *Spodoptera littoralis* | SlitOR3 | (E)-4,8-dimethylnona-1,3,7-triene | *Drosophila* OSNs | (43) |  |  |  |
|  | SlitOR4 | (±)-linalool | *Drosophila* OSNs | (43) |  |  |  |
|  | SlitOR5 | Z9,E11-14:OAc | *Drosophila* OSNs |  | Male | qPCR |  |
|  | SlitOR6 | Z9,E12-14:OAc | *Drosophila* OSNs | (44) | Male | qPCR | (44) |
|  | SlitOR7 | sulcatone | *Drosophila* OSNs | (43) |  |  |  |
|  | SlitOR13 | Z9,E12-14:OAc | *Drosophila* OSNs | (45) | Male | qPCR | (45) |
|  | SlitOR28 | Z3-hexenyl acetate | *Drosophila* OSNs | (43) |  |  |  |
|  | SlitOR35 | 3-carene | *Drosophila* OSNs | (43) |  |  |  |
|  | SlitOR36 | benzyl alcohol | *Drosophila* OSNs | (43) |  |  |  |
| *Spodoptera litura* | SlituOR6 | Z9,E12-14:OAc | *Xenopus* oocytes | (46) | Male | qPCR | (46) |
|  | SlituOR12 | Z3-hexenyl acetate | *Xenopus* oocytes | (47) |  |  |  |
|  | SlituOR13 | Z9-14:OAc; Z9,E12-14:OAc | *Xenopus* oocytes | (46) | Male | qPCR | (46) |
|  | SlituOR16 | Z9-14:OH | *Xenopus* oocytes | (46) |  |  |  |

**Supplementary references**

1. Gu SH, Sun L, Yang RN, Wu KM, Guo YY, Li XC, et al. Molecular characterization and differential expression of olfactory genes in the antennae of the black cutworm moth Agrotis ipsilon. PLoS One. 2014;9(8):e103420.

2. Zhang DD, Lofstedt C. Functional evolution of a multigene family: orthologous and paralogous pheromone receptor genes in the turnip moth, Agrotis segetum. PLoS One. 2013;8(10):e77345.

3. Zhang DD, Wang HL, Schultze A, Fross H, Francke W, Krieger J, et al. Receptor for detection of a Type II sex pheromone in the winter moth Operophtera brumata. Sci Rep. 2016;6:18576.

4. Forstner M, Breer H, Krieger J. A receptor and binding protein interplay in the detection of a distinct pheromone component in the silkmoth Antheraea polyphemus. Int J Biol Sci. 2009;5(7):745-57.

5. Xu P, Garczynski SF, Atungulu E, Syed Z, Choo YM, Vidal DM, et al. Moth sex pheromone receptors and deceitful parapheromones. PLoS One. 2012;7(7):e41653.

6. Dong J, Song Y, Li W, Shi J, Wang Z. Identification of Putative Chemosensory Receptor Genes from the Athetis dissimilis Antennal Transcriptome. PLoS One. 2016;11(1):e0147768.

7. Sakurai T, Nakagawa T, Mitsuno H, Mori H, Endo Y, Tanoue S, et al. Identification and functional characterization of a sex pheromone receptor in the silkmoth *Bombyx mori*. Proc Natl Acad Sci U S A. 2004;101(47):16653-8.

8. Wanner KW, Anderson AR, Trowell SC, Theilmann DA, Robertson HM, Newcomb RD. Female-biased expression of odourant receptor genes in the adult antennae of the silkworm, Bombyx mori. Insect Mol Biol. 2007;16(1):107-19.

9. Nakagawa T, Sakurai T, Nishioka T, Touhara K. Insect sex-pheromone signals mediated by specific combinations of olfactory receptors. Science. 2005;307(5715):1638-42.

10. Tanaka K, Uda Y, Ono Y, Nakagawa T, Suwa M, Yamaoka R, et al. Highly selective tuning of a silkworm olfactory receptor to a key mulberry leaf volatile. Curr Biol. 2009;19(11):881-90.

11. Jia XJ, Wang HX, Yan ZG, Zhang MZ, Wei CH, Qin XC, et al. Antennal transcriptome and differential expression of olfactory genes in the yellow peach moth, Conogethes punctiferalis (Lepidoptera: Crambidae). Sci Rep. 2016;6:29067.

12. Steinwender B, Thrimawithana AH, Crowhurst RN, Newcomb RD. Pheromone receptor evolution in the cryptic leafroller species, Ctenopseustis obliquana and C. herana. J Mol Evol. 2015;80(1):42-56.

13. Walker WB, 3rd, Gonzalez F, Garczynski SF, Witzgall P. The chemosensory receptors of codling moth Cydia pomonella-expression in larvae and adults. Sci Rep. 2016;6:23518.

14. Cattaneo AM, Gonzalez F, Bengtsson JM, Corey EA, Jacquin-Joly E, Montagne N, et al. Candidate pheromone receptors of codling moth Cydia pomonella respond to pheromones and kairomones. Sci Rep. 2017;7:41105.

15. Zhang SF, Zhang Z, Kong XB, Wang HB, Liu F. Dynamic Changes in Chemosensory Gene Expression during the Dendrolimus punctatus Mating Process. Front Physiol. 2018;8:1127.

16. Mitsuno H, Sakurai T, Murai M, Yasuda T, Kugimiya S, Ozawa R, et al. Identification of receptors of main sex-pheromone components of three Lepidopteran species. Eur J Neurosci. 2008;28(5):893-902.

17. Li ZQ, Luo ZX, Cai XM, Bian L, Xin ZJ, Liu Y, et al. Chemosensory Gene Families in Ectropis grisescens and Candidates for Detection of Type-II Sex Pheromones. Front Physiol. 2017;8:953.

18. Jordan MD, Anderson A, Begum D, Carraher C, Authier A, Marshall SD, et al. Odorant receptors from the light brown apple moth (Epiphyas postvittana) recognize important volatile compounds produced by plants. Chem Senses. 2009;34(5):383-94.

19. Corcoran JA, Jordan MD, Thrimawithana AH, Crowhurst RN, Newcomb RD. The Peripheral Olfactory Repertoire of the Lightbrown Apple Moth, Epiphyas postvittana. PLoS One. 2015;10(5):e0128596.

20. Yuvaraj JK, Corcoran JA, Andersson MN, Newcomb RD, Anderbrant O, Lofstedt C. Characterization of Odorant Receptors from a Non-ditrysian Moth, Eriocrania semipurpurella Sheds Light on the Origin of Sex Pheromone Receptors in Lepidoptera. Mol Biol Evol. 2017;34(11):2733-46.

21. Briscoe AD, Macias-Munoz A, Kozak KM, Walters JR, Yuan F, Jamie GA, et al. Female behaviour drives expression and evolution of gustatory receptors in butterflies. PLoS Genet. 2013;9(7):e1003620.

22. Liu Y, Liu C, Lin K, Wang G. Functional specificity of sex pheromone receptors in the cotton bollworm Helicoverpa armigera. PLoS One. 2013;8(4):e62094.

23. Di C, Ning C, Huang LQ, Wang CZ. Design of larval chemical attractants based on odorant response spectra of odorant receptors in the cotton bollworm. Insect Biochem Mol Biol. 2017;84:48-62.

24. Jiang XJ, Guo H, Di C, Yu S, Zhu L, Huang LQ, et al. Sequence similarity and functional comparisons of pheromone receptor orthologs in two closely related Helicoverpa species. Insect Biochem Mol Biol. 2014;48:63-74.

25. Wang G, Vasquez GM, Schal C, Zwiebel LJ, Gould F. Functional characterization of pheromone receptors in the tobacco budworm Heliothis virescens. Insect Mol Biol. 2011;20(1):125-33.

26. Vasquez GM, Fischer P, Grozinger CM, Gould F. Differential expression of odorant receptor genes involved in the sexual isolation of two Heliothis moths. Insect Mol Biol. 2011;20(1):115-24.

27. Yuvaraj JK, Andersson MN, Corcoran JA, Anderbrant O, Lofstedt C. Functional characterization of odorant receptors from Lampronia capitella suggests a non-ditrysian origin of the lepidopteran pheromone receptor clade. Insect Biochem Mol Biol. 2018;100:39-47.

28. Wicher D, Morinaga S, Halty-deLeon L, Funk N, Hansson B, Touhara K, et al. Identification and characterization of the bombykal receptor in the hawkmoth Manduca sexta. Journal of Experimental Biology. 2017;220(10):1781-6.

29. Koenig C, Hirsh A, Bucks S, Klinner C, Vogel H, Shukla A, et al. A reference gene set for chemosensory receptor genes of Manduca sexta. Insect Biochem Mol Biol. 2015;66:51-63.

30. Leary GP, Allen JE, Bunger PL, Luginbill JB, Linn CE, Jr., Macallister IE, et al. Single mutation to a sex pheromone receptor provides adaptive specificity between closely related moth species. Proc Natl Acad Sci U S A. 2012;109(35):14081-6.

31. Yang B, Ozaki K, Ishikawa Y, Matsuo T. Identification of candidate odorant receptors in Asian corn borer Ostrinia furnacalis. PLoS One. 2015;10(3):e0121261.

32. Liu W, Jiang XC, Cao S, Yang B, Wang GR. Functional Studies of Sex Pheromone Receptors in Asian Corn Borer Ostrinia furnacalis. Frontiers in Physiology. 2018;9.

33. Miura N, Nakagawa T, Tatsuki S, Touhara K, Ishikawa Y. A male-specific odorant receptor conserved through the evolution of sex pheromones in Ostrinia moth species. Int J Biol Sci. 2009;5(4):319-30.

34. Wanner KW, Nichols AS, Allen JE, Bunger PL, Garczynski SF, Linn CE, et al. Sex pheromone receptor specificity in the European corn borer moth, Ostrinia nubilalis. PLoS One. 2010;5(1):e8685.

35. Miura N, Nakagawa T, Touhara K, Ishikawa Y. Broadly and narrowly tuned odorant receptors are involved in female sex pheromone reception in Ostrinia moths. Insect Biochem Mol Biol. 2010;40(1):64-73.

36. Steinwender B, Thrimawithana AH, Crowhurst R, Newcomb RD. Odorant Receptors of the New Zealand Endemic Leafroller Moth Species Planotortrix octo and P. excessana. PLoS One. 2016;11(3):e0152147.

37. Liu Y, Liu Y, Jiang X, Wang G. Cloning and functional characterization of three new pheromone receptors from the diamondback moth, Plutella xylostella. J Insect Physiol. 2018;107:14-22.

38. Sun M, Liu Y, Walker WB, Liu C, Lin K, Gu S, et al. Identification and characterization of pheromone receptors and interplay between receptors and pheromone binding proteins in the diamondback moth, Plutella xyllostella. PLoS One. 2013;8(4):e62098.

39. Zhang YN, Zhang J, Yan SW, Chang HT, Liu Y, Wang GR, et al. Functional characterization of sex pheromone receptors in the purple stem borer, Sesamia inferens (Walker). Insect Mol Biol. 2014;23(5):611-20.

40. Liu C, Liu Y, Guo M, Cao D, Dong S, Wang G. Narrow tuning of an odorant receptor to plant volatiles in Spodoptera exigua (Hubner). Insect Mol Biol. 2014;23(4):487-96.

41. Du LX, Liu Y, Zhang J, Gao XW, Wang B, Wang GR. Identification and characterization of chemosensory genes in the antennal transcriptome of Spodoptera exigua. Comp Biochem Physiol Part D Genomics Proteomics. 2018;27:54-65.

42. Liu C, Liu Y, Walker WB, Dong S, Wang G. Identification and functional characterization of sex pheromone receptors in beet armyworm Spodoptera exigua (Hubner). Insect Biochem Mol Biol. 2013;43(8):747-54.

43. de Fouchier A, Walker WB, 3rd, Montagne N, Steiner C, Binyameen M, Schlyter F, et al. Functional evolution of Lepidoptera olfactory receptors revealed by deorphanization of a moth repertoire. Nat Commun. 2017;8:15709.

44. Montagne N, Chertemps T, Brigaud I, Francois A, Francois MC, de Fouchier A, et al. Functional characterization of a sex pheromone receptor in the pest moth Spodoptera littoralis by heterologous expression in Drosophila. Eur J Neurosci. 2012;36(5):2588-96.

45. de Fouchier A, Sun X, Monsempes C, Mirabeau O, Jacquin-Joly E, Montagné N. Evolution of two receptors detecting the same pheromone compound in crop pest moths of the genus Spodoptera. Frontiers in Ecology and Evolution. 2015;3.

46. Zhang J, Yan S, Liu Y, Jacquin-Joly E, Dong S, Wang G. Identification and functional characterization of sex pheromone receptors in the common cutworm (Spodoptera litura). Chem Senses. 2015;40(1):7-16.

47. Zhang J, Liu CC, Yan SW, Liu Y, Guo MB, Dong SL, et al. An odorant receptor from the common cutworm (Spodoptera litura) exclusively tuned to the important plant volatile cis-3-hexenyl acetate. Insect Mol Biol. 2013;22(4):424-32.
