# Supplementary material for: A novel lineage of candidate pheromone receptors for sex communication in moths
Source: eLife. 2019 Dec 10;8:e49826. doi: 10.7554/eLife.49826 (PMC6904214; doi:10.7554/eLife.49826)
Supplement: Transparent reporting form [file elife-49826-transrepform.docx]

***eLife’s* transparent reporting form**

We encourage authors to provide detailed information *within their submission* to facilitate the interpretation and replication of experiments. Authors can upload supporting documentation to indicate the use of appropriate reporting guidelines for health-related research (see [EQUATOR Network](http://www.equator-network.org/%2520)), life science research (see the [BioSharing Information Resource](https://biosharing.org/)), or the [ARRIVE guidelines](http://www.plosbiology.org/article/info:doi/10.1371/journal.pbio.1000412) for reporting work involving animal research. Where applicable, authors should refer to any relevant reporting standards documents in this form.

If you have any questions, please consult our Journal Policies and/or contact us: [editorial@elifesciences.org](mailto:editorial@elifesciences.org).

**Sample-size estimation**

- You should state whether an appropriate sample size was computed when the study was being designed
- You should state the statistical method of sample size computation and any required assumptions
- If no explicit power analysis was used, you should describe how you decided what sample (replicate) size (number) to use

Please outline where this information can be found within the submission (e.g., sections or figure legends), or explain why this information doesn’t apply to your submission:

For electrophysiology, behavioral and RT-qPCR experiments, sample size was not computed using an explicit power analysis. We used a standard number of replicates, commonly used for this kind of experiments (see detail in next section).

**Replicates**

- You should report how often each experiment was performed
- You should include a definition of biological versus technical replication
- The data obtained should be provided and sufficient information should be provided to indicate the number of independent biological and/or technical replicates
- If you encountered any outliers, you should describe how these were handled
- Criteria for exclusion/inclusion of data should be clearly stated

Please outline where this information can be found within the submission (e.g., sections or figure legends), or explain why this information doesn’t apply to your submission:

The RT-qPCR (Fig. 1A) was performed using 3 biological replicates and 3 technical replicates, as recommended (Bustin et. al, 2009). This has been detailed in the section “material & methods”. For electrophysiology and behavioral experiments (Fig. 2 and 3), the number of replicates is indicated in figure legends. These numbers correspond to biological replicates. Each recorded sensillum or antenna belonged to a different individual, and each trace of Two-Electrode Voltage Clamp (TEVC) was obtained from a distinct oocyte expressing the receptor of interest. For all these experiments, no recording was discarded for the calculation of mean responses.

**Statistical reporting**

- Statistical analysis methods should be described and justified
- Raw data should be presented in figures whenever informative to do so
- (typically when N per group is less than 10)
- For each experiment, you should identify the statistical tests used, exact values of N, definitions of center, methods of multiple test correction, and dispersion and precision measures (e.g., mean, median, SD, SEM, confidence intervals; and, for the major substantive results, a measure of effect size (e.g., Pearson's r, Cohen's d)
- Report exact p-values wherever possible alongside the summary statistics and 95% confidence intervals. These should be reported for all key questions and not only when the p-value is less than 0.05.

Please outline where this information can be found within the submission (e.g., sections or figure legends), or explain why this information doesn’t apply to your submission:

(For large datasets, or papers with a very large number of statistical tests, you may upload a single table file with tests, Ns, etc., with reference to sections in the manuscript.)

Statistical tests used are indicated both in the material and methods section and in the figure legends. *P*-values thresholds shown in the figures are also clearly indicated in each figure legend. All the tests used are classically used in such experiments and their choice has thus not been justified in detail. For one-way ANOVA tests, homoscedasticity of the variances has been verified first.

For phylogenies, node support estimation method was described in the material and methods section (p. 18). We used a recently published method (Lemoine et al., 2018) that has been designed for datasets with highly divergent sequences.

**Group allocation**

- Indicate how samples were allocated into experimental groups (in the case of clinical studies, please specify allocation to treatment method); if randomization was used, please also state if restricted randomization was applied
- Indicate if masking was used during group allocation, data collection and/or data analysis

Please outline where this information can be found within the submission (e.g., sections or figure legends), or explain why this information doesn’t apply to your submission:

For the RT-qPCR, animals were randomly allocated into 3 distinct groups of each sex for RNA extraction (detailed in material & methods).

For electrophysiology experiments, odors were tested in a random order except for the major pheromone component (Z9,E11-14:OAc) when tested on *S. littoralis* OSNs. It was always tested last to avoid short‐term peripheral sensitization (López, 2017).

**Additional data files (“source data”)**

- We encourage you to upload relevant additional data files, such as numerical data that are represented as a graph in a figure, or as a summary table
- Where provided, these should be in the most useful format, and they can be uploaded as “Source data” files linked to a main figure or table
- Include model definition files including the full list of parameters used
- Include code used for data analysis (e.g., R, MatLab)
- Avoid stating that data files are “available upon request”

Please indicate the figures or tables for which source data files have been provided:

Source data files have been provided for Fig. 1A-B, Fig. 2A-B-C-E, Fig.3B-C-D-E and Fig.4.
